# Nrf2, a Potential Therapeutic Target against Oxidative Stress in Corneal Diseases

**DOI:** 10.1155/2017/2326178

**Published:** 2017-10-25

**Authors:** Xiu-Fen Liu, Dan-Dan Zhou, Tian Xie, Tayyab Hamid Malik, Cheng-Bo Lu, Hai-Jun Li, Fan Wang, Chang Shu, Cong Liu, Cheng-Wei Lu, Ji-Long Hao

**Affiliations:** ^1^Department of Ophthalmology, The First Hospital of Jilin University, Jilin, China; ^2^Department of Radiology, The First Hospital of Jilin University, Jilin, China; ^3^Department of Neurosurgery, The People's Hospital of Jilin Province, Jilin, China; ^4^Department of Gastroenterology, The First Hospital of Jilin University, Jilin, China; ^5^Department of Cardiology, The First Hospital of Jiamusi University, Heilongjiang, China; ^6^Translational Medicine Research Institute, The First Hospital of Jilin University, Jilin, China; ^7^Department of Obstetrics and Gynecology, The First Hospital of Jilin University, Jilin, China

## Abstract

Corneal diseases are one of the major causes of blindness worldwide. Conservative medical agents, which may prevent sight-threatening corneal disease progression, are urgently desired. Numerous evidences have revealed the involvement of oxidative stress in various corneal diseases, such as corneal wound healing and Fuchs endothelial corneal dystrophy (FECD). Nuclear factor (erythroid-derived 2)-like 2 (Nrf2)/Kelch-like erythroid-cell-derived protein with CNC homology- (ECH-) associated protein 1 (Keap1)/antioxidant response element (ARE) signaling is well known as one of the main antioxidative defense systems. To the best of our knowledge, this is the first review to elucidate the different expression profiles of Nrf2 signaling as well as the underlying mechanisms in corneal diseases, implicating that Nrf2 may serve as a potentially promising therapeutic target for corneal diseases.

## 1. Introduction

The cornea is the transparent front part of the eye and contributes estimated two-thirds of the optical power. Human cornea is mainly composed of corneal epithelium (the outer layer), stroma (the middle layer), and endothelium (the inner layer). Normal morphologies and functions of these cells maintain the transparency of the cornea. The ocular surface mucosa (mainly the cornea) is the first layer of the eye that is exposed to environmental stress. The cornea is susceptible to be damaged by varieties of external stresses due to its constantly direct exposure to harmful factors, such as physical or chemical injuries, UV radiation, and air pollutants (gases, vapors, or cigarette smoke). Oxidative stress is characterized by the generation of reactive oxygen species (ROS), which contains superoxide anion (O_2_^−^), hydrogen peroxide (H_2_O_2_), and hydroxyl radical (^•^OH), and is considered to be involved in these external stresses [1]. Under normal condition, ROS production and cleavage is counterbalanced.

Nuclear factor (erythroid-derived 2)-like 2 (Nrf2 or NFE2L2), encoded by the gene of NFE2L2, is a vital nuclear transcription factor for the systemic antioxidant defense system. In basal conditions, Nrf2 binds to Kelch-like erythroid-cell-derived protein with CNC homology- (ECH-) associated protein 1 (Keap1) as a complex and is restricted to the cytoplasm where it undergoes ubiquitination and proteasomal degradation. Under stressed condition, Nrf2 separates from Keap1 (a primary Nrf2 inhibitor) and is translocated into the nucleus, where it binds to the phase 2 of antioxidant response element (ARE) in the DNA promoter region and initiates the transcription of ARE controlled antioxidative enzymes, such as superoxide dismutase (SOD), catalase, glutathione S-transferase (GSTP) [2], NAD(P)H: quinone oxidoreductase 1 (NQO1) [3], heme oxygenase-1 (HO-1) [4], thioredoxin reductase (TrxR), glutathione reductase (GR), and glutathione-S-transferase (GST) [5, 6], which detoxify ROS through GSH regulation [7]. Nrf2 is known as the molecular switch turning on/off the Nrf2 signaling (also known as Nrf2-Keap1 or Nrf2-Keap1-ARE signaling) and serves as a foremost component of ROS signaling pathway that can be activated by oxidative stress inducers, such as sulforaphane (SFN) [8] and hyperoside [9], and inhibited by oxidative stress factors such as homocysteine (Hcy) [10], O_2_ fluctuation [11], and hypoglycemia under hypoxia [12].

Normally, there is a balance between the systemic generation of oxidants and biological antioxidants' capacity to remove the oxidants or to repair the oxidative stress-induced damage. Overproduction of ROS or dysfunction of antioxidative enzymes can result in oxidative stress and lead to cellular damages (e.g., lipid peroxidation of cell membranes and oxidative damage to DNA and proteins). Oxidative stress is known as a vital pathogenesis underlying aging and many human ocular diseases, such as corneal diseases (injuries, keratoconus, Fuchs' endothelial dystrophy (FECD), etc.), dry eyes, cataracts, glaucoma, age-related macular degeneration, and other oxidative-related diseases in the eyes [1]. The protective role of Nrf2 against oxidative stress in ocular surface diseases has been clarified in the following researches: sidestream cigarette smoke (SCS) exposure-induced dry eyes [13] and pterygium [14, 15].

To the best of our knowledge, this is the first review to elucidate the specific role and the underlying mechanism of Nrf2-mediated antioxidative defense in corneal diseases, including wound healing, Fuchs' endothelial dystrophy, and corneal regenerative projects.

## 2. Nrf2 in Corneal Diseases

### 2.1. Nrf2 in Corneal Wound Healing

Located in the foremost layer of the cornea, corneal epithelial cells are easily injured by external factors such as physical injuries, chemical injuries, and oxidative stress (UV radiation). Delayed corneal wound healing is often found in the cornea of diabetic patients. It was revealed that the topical application of a high dose of carnosol (1 mM), a well-established Nrf2 activator, accelerated the corneal wound healing in diabetic rats with the corneal epithelium mechanically removed [16]. A Nrf2-Keap1-dependent protective role against oxidative stress in wound healing was also clarified in a heptanol-induced corneal epithelial wound model. The results found that Nrf2 was activated both in the preinjured and postinjured corneal epithelium of wild-type (WT) mice, indicating the involvement of Nrf2 throughout the corneal healing process [17]. Corneal epithelial cells' migration and wound healing were significantly delayed in Nrf2 knockout (KO) mice than those of WT mice, accompanied by the detection of Nrf2 activation and then translocation to the nuclei via immunostaining. However, the corneal cell proliferation was not affected in both KO and WT mice, demonstrated by the immunostaining for Ki-67 (a proliferative marker) [17]. Nrf2 knockdown was performed by siRNA in a corneal epithelial cell line (C/TERT) in vitro to further elucidate the role of Nrf2 during the corneal wound healing, and the results revealed that Nrf2 siRNA significantly delayed C/TERT cell migration (but not proliferation), which is accompanied by the decreased transcriptions of Nrf2-dependent antioxidative genes (HO-1 and NQO1). On the contrary, Keap1 siRNA dramatically accelerated C/TERT cell migration with increased HO-1 and NQO1 expressions [17]. All these studies support the concept that Nrf2 plays a protective role in corneal epithelial wound healing, mainly by accelerating cell migration via the initiation of Nrf2-mediated antioxidative defense system. And the exact mechanisms of Nrf2 regulating corneal epithelial cell migration need to be clarified; Nrf2 would serve as a promising target for the treatment of corneal wound healing.

Corneal keratocytes are activated and transformed into myofibroblasts and fibroblasts upon injury, which is an important biological event during corneal wound repair with scar formation [18]. Ethyl pyruvate (EP), a pyruvate ester which augments pyruvate levels, possesses the ability to ameliorate the cellular oxidative stress [19]. The Nrf2-mediated antioxidant response was enhanced by EP in keratocytes and myofibroblasts and induced phenotypic changes of inactive corneal stromal keratocytes into contractile myofibroblasts cultured in vitro, indicating the therapeutic potential of EP in corneal wound healing [20]. Trichostatin A (TSA), a nonselective inhibitor of histone deacetylase (HDAC), was revealed to possess the capacity for fibrosis prevention [21]. It was reported that treatment with TSA showed a solid protective effect against the oxidative stress induced by transforming growth factor-*β* (TGF-*β*) and a strong inhibition of myofibroblast differentiation in TGF-*β*-stimulated human immortalized corneal fibroblasts cultured in vitro. In addition, TSA decreases ROS and H_2_O_2_ accumulation, persuades Nrf2 nuclear translocation, and upregulates the transcriptions of Nrf2-ARE-controlled antioxidant enzymes (such as GSH). In opposite, Nrf2 siRNA prevented the inhibitory effect of TSA on TGF-*β*-induced myofibroblast differentiation. These convincing results implicated that Nrf2 was involved in myofibroblast differentiation, and TSA may serve as a promising medical alternative preventing corneal wound scar formation, via inhibiting Nrf2-ARE-regulated myofibroblast differentiation [22].

### 2.2. 4-Hydroxynonenal and Nrf2 in Corneal Epithelial Disease

**Figure 1:**
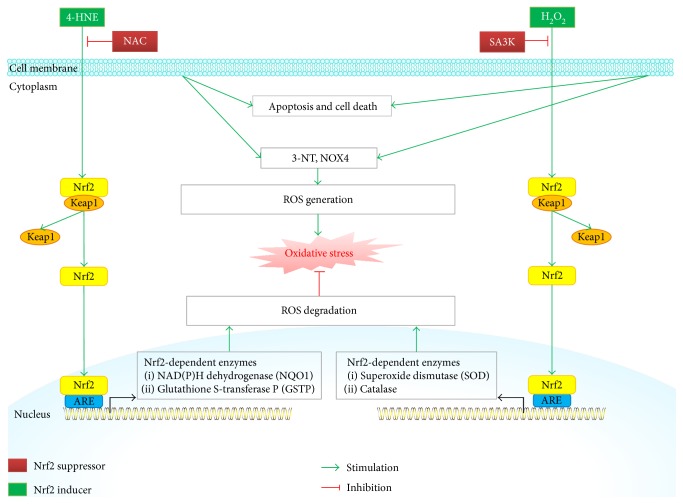
Schematic diagram of Nrf2 signaling and regulation in the corneal epithelial cells. 4-HNE or H_2_O_2_ induces excessive ROS generation (by upregulation of 3-NT, NOX4 protein expression), leading to oxidative stress in the cultured HCE cells and resulting in cell apoptosis and death of corneal epithelial cells. 4-HNE activates Nrf2/ARE-controlled antioxidant enzyme (NQO1 and GSTP) transcription, which facilitates ROS degradation. On the other hand, H_2_O_2_ decreases Nrf2/ARE-controlled SOD and catalase transcription, leading to ROS degradation suppression. NAC serves as an antioxidant by antagonizing 4-HNE overexpression and reversing the cell viability of HCE cells. SA3K blocks H_2_O_2_-induced ROS, 3-NT, and NOX4 overexpression and upregulates ROS degradation by activating Keap1-Nrf2-ARE pathway.

4-Hydroxynonenal (4-HNE), a major endogenous product of lipid peroxidation and a key marker of oxidative stress, is considered to play oxidant roles in corneal diseases, such as Fuchs endothelial corneal dystrophy (FECD) and keratoconus [23]. 4-HNE was found to inhibit the cell viability by increasing the level of 3-nitrotyrosine (3-NT, a marker of oxidative stress) and NADPH oxidase 4 (NOX4, a vital enzyme of ROS generation) in cultured human corneal epithelial (HCE) cells via Western blot and immunofluorescent staining [24]. 4-HNE also increased the cytoplasmic expression and nuclear translocation of Nrf2 as well as the transcription of Nrf2-dependent effectors: GSTP and NQO1 in cultured HCE cells [24], indicating that 4-HNE can induce oxidative stress in the corneal epithelium through the activation of Nrf2 and its effectors. N-Acetylcysteine (NAC), a classic ROS scavenger, antagonized the 4-HNE-induced oxidant effects in the cultured HCE cells, evidenced by the reversed cell viability of HCE cells, and reduced the 3-NT, NOX4, and Nrf2 protein expression induced by 4-HNE (Figure 1) [24]. These results elucidated the relationships among the lipid peroxidation, oxidative stress, and antioxidative defense in the corneal epithelium, providing a potential therapeutic direction for oxidative-related eye diseases. Further intensive studies are still needed to explore the full mechanisms of 4-HNE-induced oxidative stress in corneal epithelium, such as the binding site and other targeting factors.

### 2.3. H_2_O_2_ and Nrf2 in Corneal Epithelial Disease

H_2_O_2_ is a main ROS product and known as an oxidative stressor in experimental researches. Various factors, such as Keap1-Nrf2 pathway and NOX4 (an isoform of NADPH oxidase), are considered to be involved in the complex process of oxidative stress. SERPINA3K (SA3K) belongs to the serine proteinase inhibitor family and possesses antioxidant effect. It prevented against H_2_O_2_-triggered apoptosis and ROS overproduction as well as repressed GSTP and NQO1 in cultured HCE cells [2]. Meanwhile, it accelerated ROS degradation by upregulating the activity of antioxidant ROS degradation enzymes, such as catalase and superoxide dismutase. In agreement with in vitro study, SA3K is also demonstrated to protect rat corneal epithelium in vivo against oxidative stress by inhibiting ROS generation and suppressing the Keap1-Nrf2 pathway and its downstream factor NOX4 (Figure 1) [2]. Taken together, SA3K protects against H_2_O_2_-induced oxidative stress in corneal epithelium by restoring the balance between ROS generation and degradation, as well as regulating the Keap1-Nrf2 signaling pathway, indicating that SA3K is a promising antioxidant factor that may serve as a potential therapeutic agent for the oxidative stress-related corneal diseases. Further and in-depth studies based on the transgenic animal models (e.g., transgenic Tg-SOD mice), which may elucidate the comprehensive mechanisms of SA3K on the Keap1-Nrf2 system, such as SA3K's binding site, the specific pathway (e.g., Wnt pathway) involved in between SA3K and Keap1-Nrf2-ARE system, are needed.

### 2.4. Role of Nrf2 in Corneal Regeneration via Stem Cells

Under normal circumstance, the injured corneal epithelium is regenerated by the corneal limbal stem cells. The physiological regenerative function of stem cells is lost in corneal limbal stem cell deficiency (LSCD), leading to corneal opacity and vision impairment. Transplantation of stem cells is applied to treat LSCD, using tissue-engineered epithelial cell sheets, such as human oral mucosal epithelial cell (hOEC) sheets [25, 26] and human induced pluripotent (iPS) stem cells [27]. These stem cell-based regenerative cell sheet techniques facilitate the regeneration of the patient's own damaged stem cells and showed positive effects in treating LSCD in clinical trials [28]. A healthy status of stem cells in tissues is the foremost factor in regenerative medicine, and feasible preservation techniques, which may improve the preservation for the cell sheet, are required due to the merging stem cell-based application and transplantation.

Ebselen, an organic selenium-containing redox compound and a well-known Nrf2 activator, has exhibited great potentials as a promising medium for the preservation of tissue-engineered cell sheets and the stem/progenitor cells under hypothermia during preservation. The expression of two tight junction-relevant proteins (ZO-16 and MUC165), which maintain the barrier function of the corneal epithelium, were enhanced, and the reduction of p63 (an epithelial stem cell marker) was prevented by Ebselen in the hOEC sheet under hypothermic stress [25]. Ebselen also maintained the high ATP levels, normal morphology, viability, and function of the hOEC sheet during hypothermic preservation by reducing ROS generation, inducing the Nrf2 activation, decreasing the lactate dehydrogenase (LDH) releasement, and increasing the GSH/oxidized glutathione (GSSG) ratio. Ebselen-initiated Nrf2 activation exerts the antioxidant, as well as the antiapoptotic, effect which protects the cells against oxidative stress-triggered damage caused by the hypothermia during preservation. Though not evidenced during preservation, Nrf2 translocation induced by ebselen in the hOEC sheets was evidenced after 3–6 hours of reculturing at 37°C. These results indicate that ebselen possesses two different biological roles: one is the direct effect of removing hypothermic-induced ROS generation during the preservation period and the other is the indirect effect via Nrf2 translocation during the reculturing period [25]. Moreover, ebselen maintained the normal morphology of limbal epithelial layer and showed meaningfully higher colony-forming efficiency, when the human corneal limbal tissue was preserved in ebselen, when compared to that of control in which a great number of corneal epithelium was lost [25]. Therefore, ebselen was demonstrated to be an effective hypothermic preservation medium for tissue-engineered cell sheets, and it is believed to promote the ocular regenerative medicine, such as LSCD management and cornea transplantation.

It was also reported that murine corneal epithelial progenitor cell line (TKE2) is more resistant to H_2_O_2_-induced oxidative stress with enhanced atrophy than cultured mature murine corneal epithelial cells (MCE). It was regulated by decreasing ROS production, reducing oxidative enzymes, such as NADPH oxidase 4 (NOX4), and increasing dual specificity phosphatase 6 (DUSP6). TKE2 also activated Nrf2 signaling and upregulated the expression of antioxidative enzymes (SOD and GSTP), indicating that Nrf2 is involved in maintaining the different hemostasis of corneal stem cells and exhibits strong antioxidant capacity against oxidative stress by regulating ROS generation and elimination [25].

### 2.5. Nrf2-Regulated Antioxidant Defense and Corneal Endothelial Dystrophy

FECD is a blinding disease and a primary reason of endogenous corneal endothelial degeneration. It is a gradually progressing disease with the accumulation of extracellular excrescences (guttae) [29]. Corneal transplantation is the only method to restore lost vision in patients with FECD. Jurkunas et al. reported that the imbalance of oxidant-antioxidant is indispensable in the long-lasting deteriorating progression of corneal endothelium (CE) observed in FECD [30]. Nrf2, a vital nuclear transcriptional inducer, which binds to the ARE in the DNA promoter and initiates antioxidant defense, is downregulated in FECD endothelium. Higher levels of oxidative DNA damage and apoptosis of CE were also detected in FECD endothelium in contrast to controls [30]. 8-Hydroxy-2-deoxyguanosine (oxidative DNA damage marker) localized to the mitochondria, demonstrating that the genome of the mitochondria is the main target of oxidative stress in FECD. This study revealed that oxidative stress plays a vital role in FECD pathogenesis.

#### 2.5.1. Ultraviolet A and Nrf2-Regulated Antioxidant Defense in FECD

**Figure 2:**
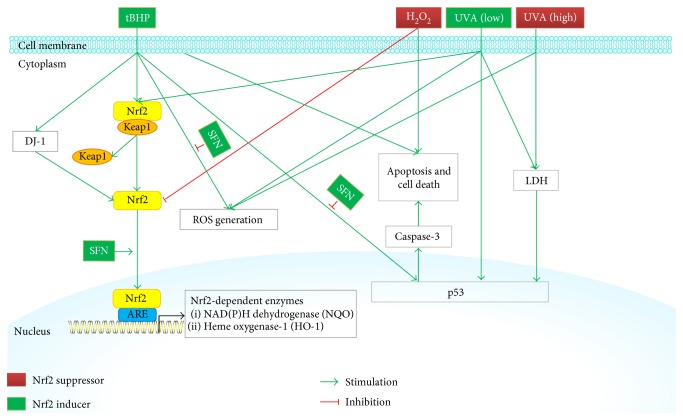
Schematic diagram of Nrf2 signaling and regulation in the corneal endothelial cells. TBHP and H_2_O_2_, both high and low fluences of UVA, can induce excessive ROS generation in corneal endothelial cells, leading to apoptosis and cell death. SFN can inhibit tBHP-induced ROS generation, apoptosis, and cell death. Meantime, it can increase DJ-1 protein expression and Nrf2 translocation and induce Nrf2/ARE-dependent antioxidant enzyme (HO-1 and NQO1) transcriptions.

Ultraviolet A (UV-A) with the wavelengths of 320 to 400 nm is the main source of solar radiation, which plays an important role in ROS production, and therefore may be related to the etiology of FECD. The cornea is radiated daily by solar ultraviolet (UV) rays, which can lead to DNA damage and induce oxidative stress [31]. Jurkunas et al. reported that lower fluences of UV-A activated the antioxidant defense regulated by Nrf2 and higher fluences initiated p53 and caspase-3, denoting that near-environmental fluences of UV-A may have influence on normal human corneal endothelial cells (CECs) (Figure 2()). Other studies have also indicated the key role of Nrf2-mediated antioxidant defense as well as p53 in the regulation of oxidative stress-induced apoptosis in FECD [30, 32, 33]. Those studies can introduce an in vitro oxidative stress model for exploring CEC degeneration, particularly, in FECD pathogenesis.

#### 2.5.2. DJ-1 and Nrf2-Regulated Antioxidant Defense in FECD

DJ-1, encoded by the PARK7 gene, is a multifunctional protein and is universally expressed in most human cells and tissues. Under the influence of oxidative stress, DJ-1 plays an important role in antioxidant defense by regulating several antioxidant gene expressions [34], as a ROS scavenger [35], and suppresses proapoptotic factors, which guard against oxidative stress- and UV-stimulated cell apoptosis [36]. It is suggested that DJ-1 prevents the Keap1/Nrf2 linkage [37, 38] or nuclear export of Nrf2 [39] to stabilize the function of Nrf2. It is reported that DJ-1 reduced drastically in FECD CECs, while Keap1 (Nrf2 protein repressor) increased under oxidative stress [33]. Nrf2 nuclear localization was detected in normal CECs, whereas the translocation of Nrf2 from cytoplasm to nuclei was not observed in FECD. Decreased levels of DJ-1 in FECD at baseline and under the stressed state were in relation with weakened Nrf2 nuclear translocation and improved cell susceptibility to apoptosis. The DJ-1/Nrf2 axis could be a promising target to delay the degeneration of CECs in FECD [33]. Nevertheless, these opinions are opposed by a report that DJ-1 is not involved in triggering the Nrf2-ARE signal pathway [40]. Recently, Liu et al. further proved that downregulation of DJ-1 resulted in decreased Nrf2 gene expression as well as its target genes HO-1 and NQO1 (Figure 2), which inhibits translocation of Nrf2, leading to attenuate the expression of antioxidant gene and oxidative damage [41]. The decrease of DJ-1 level results in enhanced CEC susceptibility to UV-A light via inducing p53-dependent apoptosis [41]. Therefore, focusing on the DJ-1/Nrf2 axis may offer a prospective treatment of corneal endothelial disorders through improving antioxidant defense.

#### 2.5.3. SFN and Nrf2-Regulated Antioxidant Defense in FECD

SFN is an Nrf2 level enhancer, which is found in green cruciferous vegetables like broccoli [42]. Antioxidative stress effects of SFN have been widely studied [43, 44]. SFN and tBHP have been found to initiate modification of Keap1/Nrf2 proteins, leading to the activation of Nrf2 [33, 45]. Recently, Ziaei et al. had reported that SFN increased the activation of Nrf2 in FECD specimens and reduced p53 staining under oxidative stress. Pretreatment with SFN improved cell viability via diminishing the production of intracellular ROS in FECD. Increased level of Nrf2 resulted in unregulated synthesis of HO-1, DJ-1, and oxidoreductase [46]. Nrf2-ARE pathway may be a vital molecular mechanism involved in degenerative cell loss detected in FECD. SFN can drastically increase ARE-dependent antioxidants and reduce apoptosis induced by oxidative stress in FECD (Figure 2).

#### 2.5.4. SLC4A11 and Nrf2-Regulated Antioxidant Defense in FECD

SLC4A11 is an anion transporter and a dimer located in the plasma membrane. It encodes a 100 kDa protein containing 14 domains [47]. Lately, it has been exposed to take part in Na^+^-coupled OH_2_ transport in CECs of bovine [48]. SLC4A11 is greatly expressed in human corneal epithelial as well as endothelial cells [49]. Dysfunctional SLC4A11 is supposed to be a contributing factor of corneal endothelial cell death [50]. CHED2 is an autosomal recessive disorder, which is featured by decreasing in corneal endothelial cell density [51]. Both CHED2 and FECD result in gradual cloudiness of the cornea and progressive vision loss. Some studies have reported that they have been connected with several mutations in the SLC4A11 gene [52–56], which can induce ROS generation and mitochondrial dysfunction due to oxidative stress. It is detected that 2 major Nrf2 transcriptional targets, HO-1 and NQO, as well as Nrf2 expression, declined drastically, and higher apoptosis rate was found in cells with mutant proteins in overexpressed ROS environment [4]. Further studies are needed to elucidate the specific role of SLC4A11 played in corneal endothelial dysfunction, which may facilitate the management of the corneal endothelial cell degeneration.

## 3. Conclusions and Outlook

Accumulating evidences denote that oxidative stress is one of the major mechanisms involved in the corneal diseases, which increases the injury in the corneal epithelial and endothelial cells via oxidation of proteins, DNA damage, apoptosis, cell death, and so forth. The Nrf2/Keap1/ARE signaling pathway is related to cell defense mechanisms against oxidative stresses. Therefore, the initiation of the Nrf2-ARE signaling pathway has been estimated as an important target for the design and synthesis of new agents for corneal diseases. Selected studies on the relationship between Nrf2 inducer/suppressor and corneal diseases reviewed in this article have shown notable effects on protecting or deteriorating corneal epithelial or endothelial cells against oxidative stress, decreasing the aberrant proteins and preventing or causing the corneal diseases (Table 1). All these encouraging effects have been linked with the antioxidant and Nrf2-inducing effects of the compounds studied (Figures 1 and 2). In summary, the Nrf2/Keap1/ARE signaling pathway is a promising therapeutic target against oxidative stress for corneal diseases.

## Figures and Tables

**Table 1:** Selected studies on the relationship between Nrf2 inducer/suppressor and corneal diseases.

Inducer	Suppressor	Type	Models	Results	Reference
4-HNE	NAC	In vitro	Cultured HCE cells	NAC antagonized the 4-HNE-induced oxidant effects in the cultured HCE cells by the reversed cell viability of HCE cells and reduced the 3-NT, NOX4, and Nrf2 protein expression induced by 4-HNE.	[24]
H_2_O_2_	SA3K	In vitro	Cultured HCE cells	SA3K reversed H_2_O_2_-induced cell apoptosis. SA3K upregulated H_2_O_2_-induced downregulation of SOD2 and catalase gene expression. SA3K blocked H_2_O_2_-induced ROS and NOX4 overexpression and activated Keap1-Nrf2 pathway by suppressing Keap1-Nrf2-ARE pathway.	[2]
		In vivo	Rat corneal epithelium	SA3K ameliorated H_2_O_2_-induced corneal epithelium death and decreased the H_2_O_2_-induced ROS, 3-NT, NOX4, and Nrf2 overexpression.	
	H_2_O_2_	In vitro	Cultured TKE2 and MCE	TKE2 cells have different homeostasis and strong antioxidant properties compared to MCE by decreasing ROS production and NOX4 and increasing DUSP6, Nrf2, SOD, and GSTP.	[25]
Ebselen		In vitro	hOEC sheet, human corneal limbal tissue	Ebselen maintained the high ATP levels, normal morphology, viability, and function of the hOEC sheets by reducing ROS generation, inducing the Nrf2 activation, decreasing the lactate dehydrogenase (LDH) releasement, and increasing the glutathione (GSH)/oxidized glutathione (GSSG) ratio. Ebselen maintained the normal morphology of limbal epithelial layer and showed meaningfully higher colony-forming efficiency.	[25]
Carnosol		In vivo	Diabetic rat corneal epithelium injury model	Carnosol accelerated the corneal epithelial wound healing.	[16]
EP		In vitro	Cultured keratocytes and myofibroblasts	EP enhanced the Nrf2-mediated antioxidant response and induced phenotypic changes of quiescent corneal stromal keratocytes into contractile myofibroblasts.	[20]
TSA	TGF-*β*	In vitro	Cultured corneal fibroblasts (HTK)	TSA inhibited of TGF-*β*-stimulated myofibroblast differentiation in HTK cell line, by decreasing ROS and H_2_O_2_ accumulation, inducing Nrf2 nuclear translocation and upregulated the transcriptions of Nrf2-ARE-controlled antioxidant enzymes (such as GSH).	[22]
Lower fluences of UV-A		In vitro	CECs	Lower fluences of UV-A activated the antioxidant defense regulated by Nrf2 and higher fluences initiated p53 and caspase-3. UV-A may be related to the etiology of FECD.	[57]
		In vitro	FECD endothelium	Nrf2 is downregulated in FECD endothelium; higher levels of oxidative DNA damage and apoptosis of CE were also detected in FECD endothelium in contrast with normal controls.	[30]
tBHP		In vitro	HCECi and FECDi; FECD corneal buttons	Declined levels of DJ-1 in FECD at baseline and under the condition of oxidative stress were in relation with weakened Nrf2 nuclear translocation and improved cell susceptibility to apoptosis.	[33]
UV-A		In vitro	CECs	Downregulation of DJ-1 resulted in decreased Nrf2 gene expression as well as its target genes HO-1 and NQO1, which inhibits translocation of Nrf2, leading to attenuate the expression of antioxidant gene and increase oxidative damage. The decrease of DJ-1 level results in enhanced CECs susceptibility to UV-A light via inducing p53-dependent apoptosis.	[41]
SFN		In vitro	HCECi and FECDi	SFN increased the activation of Nrf2 in FECD specimens under the condition of oxidative stress. Pretreatment with SFN improved cell viability via diminishing the production of intracellular ROS in FECD.	[46]
	SLC4A11 mutations	In vitro	HEK 293 cells	Mutations in the *SLC4A11* gene can induce ROS generation and mitochondrial dysfunction because of oxidative stress. HO-1, NQO, and NRF2 expression declined drastically, and a higher apoptosis rate was found in cells with mutant proteins under oxidative stress.	[4]

4-HNE: 4-hydroxynonenal; 3-NT: 3-nitrotyrosine; NOX4: NADPH oxidase 4; HCE: human corneal epithelial cells; GSTP: glutathione S-transferase P; NQO1: NAD(P)H dehydrogenase (quinone 1); NAC: N-acetylcysteine; H_2_O_2_: hydrogen peroxide; SA3K: SERPINA3K; TKE2: murine corneal epithelial progenitor cell line; MCE: mature murine corneal epithelial cells; NOX4: NADPH oxidase 4; DUSP6: dual specificity phosphatase 6; SOD: superoxide dismutase; GSTP: glutathione S-transferase P; EP: ethyl pyruvate; TSA: trichostatin A; TGF-*β*: transforming growth factor-*β*; CECs: corneal endothelial cells; UV-A: ultraviolet A; FECD: Fuchs endothelial corneal dystrophy; Nrf2: nuclear factor erythroid 2-related factor-2; FECDi: immortalized FECD human corneal endothelial cell lines; HCECi: immortalized normal human corneal endothelial cell lines; tBHP: tertbutyl hydroperoxide; SFN: sulforaphane; HEK 293: human embryonic kidney.
